# Self-Supervised Learning for Point-Cloud Classification by a Multigrid Autoencoder

**DOI:** 10.3390/s22218115

**Published:** 2022-10-23

**Authors:** Ruifeng Zhai, Junfeng Song, Shuzhao Hou, Fengli Gao, Xueyan Li

**Affiliations:** 1 State Key Laboratory of Integrated Optoelectronics, College of Electronic Science and Engineering, Jilin University, Changchun 130012, China; 2 Peng Cheng Laboratory, Shenzhen 518000, China

**Keywords:** 3D point-cloud classification, deep learning, self-supervised learning

## Abstract

It has become routine to directly process point clouds using a combination of shared multilayer perceptrons and aggregate functions. However, this practice has difficulty capturing the local information of point clouds, leading to information loss. Nevertheless, several recent works have proposed models that establish point-to-point relationships based on this procedure. However, to address the information loss, in this study we use self-supervised methods to enhance the network’s understanding of point clouds. Our proposed multigrid autoencoder (MA) constrains the encoder part of the classification network so that it gains an understanding of the point cloud as it reconstructs it. With the help of self-supervised learning, we find the original network improves performance. We validate our model on PointNet++, and the experimental results show that our method improves overall classification accuracy by 2.0% and 4.7% with ModelNet40 and ScanObjectNN datasets, respectively.

## 1. Introduction

Point clouds are used to accurately reflect the spatial information of objects for various computer vision tasks, such as robotics [1], autonomous vehicles [2], remote sensing [3], virtual reality, and augmented reality [4]. Point-cloud shape classification is the basis of many applications and provides semantic information to downstream tasks, which is often key to realizing system functionality.

The unordered and unoriented data structure of point clouds prevents us from directly handling the data using 2D image deep learning methods [2,5]. Through the unremitting efforts of researchers, three mainstream processing methods for point-cloud shape classification have been developed: multiview-, volumetric-, and point-based methods. Multiview and volumetric types must first convert them to projection images and voxels, respectively. The disadvantages of these methods are increased computation and information loss. However, point-based methods directly process point clouds. The research in this paper focuses on point-based methods.

Point-based methods are subdivided into pointwise multilayer perceptrons (MLPs) and convolution- and graph-based methods [6]. PointNet [7] was the first network of this type, and it is highly efficient and effective. Subsequently, a more complete version, PointNet++ [8], was proposed by Charles R. Qi. Notably, most point-based methods strongly rely on similar pointwise MLP methods. PointCNN [9] uses them to construct the X-Conv operator, and ConvPoint [10] uses them to generate spatial weights. KPConv [11], InterpConv [12], FoldingNet [13], and point transformer [14] methods use them to extract features from point clouds. To improve upon these models, it is first necessary to carefully study the principle of their methods.

The principle of pointwise methods is the sharing of MLPs to extract features, followed by symmetric aggregation to overcome the problem of unordered data [6]. However, following this principle causes a new problem, i.e., the receptive field of shared MLPs becomes too small to accurately construct the relationship between neighboring points, resulting in shape information loss. In recent years, researchers have proposed various methods to fix this. For example, PointWeb [5] introduced the adaptive feature adjustment module to PointNet++ so that neighboring point relationships can be constructed. Duan proposed the structural relation network (SRN) module to enhance the inner relations between local point-cloud structures [15]. Notably, these methods expand the receptive field by adding complex structures to the original network.

**Figure 1:**
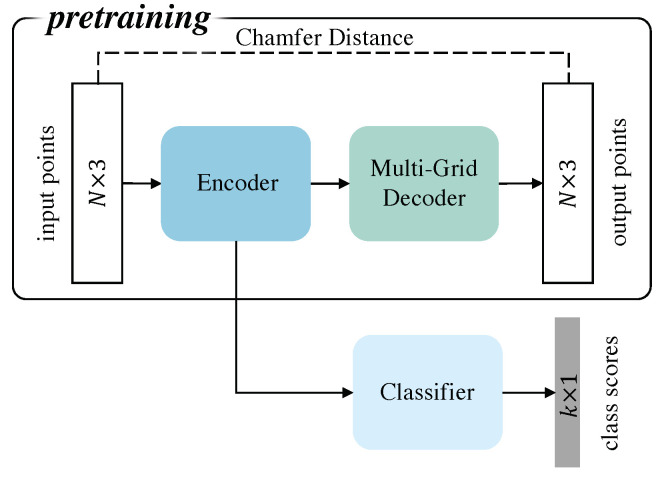
Overall self-supervised model framework. During pretraining, the upper network trains the multigrid autoencoder. When training the classification network, the lower network completes the classifier’s training and fine-tunes the overall classification network.

Inspired by the success of self-supervised, transfer, and multitask learning methods applied to 2D image deep learning networks [16,17], we propose a self-supervised structure with a multigrid autoencoder that effectively improves the classification performance of PointNet++ [8]. The method’s framework is shown in Figure 1. To enhance the ability of neural networks to understand the relationship between points, it is intuitive to introduce complex modules to the network [5,15]. The novelty of our method is the addition of shape constraints. Our proposed method first forces the neural network to learn the shape feature of the point cloud by pretraining the encoder. Then, the classifier learns the category feature, and the network is fine-tuned.

The autoencoder constrains the encoder, forcing it to learn point locations and understand; however, an autoencoder neural network that can handle them is needed. For this, we require the autoencoder’s output to be similar in shape to the original point cloud so that the geometrical and positional relationships between points can be learned. At present, there is not much research on point-cloud autoencoders, and more work focuses on the task of point-cloud reconstruction. We aim to use them with a little modification, so they must meet the following requirements: (1) their encoder can easily be replaced by a classification network; and (2) their ground truth can be easily obtained. Therefore, we investigated some point-cloud completion networks [18,19,20,21] and found that they possess strong reconstruction capabilities; however, they fall short of our requirements. Eventually, we built a multigrid autoencoder inspired by FoldingNet [13,22,23]. Unlike FoldingNet, which implements a single mesh to point-cloud mapping, a multigrid autoencoder maps multiple independent grids to the point cloud. Its encoder can be replaced by most encoders with a hierarchical structure, and the ground truth it needs is the point cloud itself.

Our main contributions are as follows:

- A self-supervised method that enhances PointNet++ classification performance.
- A pretraining process that enables the encoder to implicitly acquire the ability to extract the positional relationship of points, reducing shape information loss.
- A multigrid autoencoder that effectively extracts point-cloud shape information without supervision.
- MLP-enabled multi-scale fusion improved network performance via pretraining.

## 2. Related Work

### 2.1. Point-Cloud-Based Classification Models

Compared with multiview- and volumetric-based networks, point-based networks have the advantage of not converting point clouds to other forms. Therefore, the data obtained by these models are more accurate and actionable, reflecting the 3D geospatial and local structure information of point clouds [2].

In 2017, PointNet [7] was the first point-based network. It creatively uses shared MLPs to extract the features of each point and applies the max-pooling function to obtain the global features of the point cloud. This combination effectively overcomes the problem of point-cloud disorder. Notably, it uses the T-Net module to overcome the challenges posed by the rotation invariance of point clouds. However, PointNet is disadvantaged in that it cannot extract local features. Subsequently, PointNet++ [8] was built to solve this problem. It simplifies PointNet into its smallest module to extract the local features of point clouds, similar to convolutional 2D image processing. PointNet++ also uses multiscale and multi-resolution grouping to address the challenges of the nonuniform density of point clouds. However, this network cannot establish connections between points.

Many researchers have proposed methods to overcome this limitation. In 2019, Moment [24], e.g., is similar in structure to PointNet, but it adds polynomial functions to point-cloud coordinates so that it can compute higher-order moments. PointWeb [5] adds an adaptive feature adjustment module preceding the PointNet++ shared MLP to enhance the connections between neighboring points, enabling the extraction of richer information. In 2020, PointAugment [25] used an auto-augmentation framework for point-cloud classification to generate a shape-rich point cloud with preserved class features by rotating and changing the positions of points in the network. In contrast to these methods, we enhance the performance of the classification network using a multigrid autoencoder to force the encoder to learn the shape of the point cloud and its positional relationship.

Convolutions are widely used for 2D image deep learning [26], and many researchers have proposed such methods for use with point clouds. For example, in 2018, PointCNN [9] generated a transformation matrix using shared MLPs to improve local point-cloud sorting to enable convolution operations. In 2019, PointConv [27] implemented convolution operations by generating its own convolution kernels, which require only a small amount of memory. KPConv [11] and InterpConv [12] differ from these methods in that they estimate the corresponding value of the neighboring point at the position of the convolution kernel. However, their convolution frameworks and calculation methods differ greatly. In 2020, ConvPoint [10] generated spatial weights using shared MLPs to adjust the internal parameters of convolution kernels so that they have one-to-one correspondence with points.

The transformer has been used to enable considerable breakthroughs in natural language [28] and 2D image processing [29]. In 2021, the point transformer method [14] used a point transformer block with transition-down and -up modules to process point clouds, and the point-cloud transformer (PCN) of [30] used the transformer’s permutation invariance to classify and segment point clouds.

### 2.2. Self-Supervised Learning Models for Point Clouds

Self-supervised learning has achieved remarkable results in many domains [16,31,32], as it avoids tedious manual annotations. Presently, most methods first apply a pretext task to learn valuable semantic information, and it then fine-tunes the weight of the network to achieve the target task.

In 2019, the pretext task of ContrastNet [33] determined whether two incomplete point clouds are of the same category. Kaveh Hassani designed an unsupervised multitask model [34]. These tasks are reconstruction, clustering, and pseudo-label-based prediction types. The pretext task in shape self-correction [35] restores the disturbed part of the point cloud. The original intention of these self-supervised methods was to hope that the encoder could learn more semantic features of the point cloud. However, our original intention is that the encoder can learn both semantic features and local features between points. In 2021, OcCo [36] applied a self-supervised learning method for point clouds consisting of three steps. First, a point cloud with occlusion is generated according to the camera’s perspective; then, the autoencoder uses the data to complete the task. Finally, the autoencoder’s weights are fine-tuned to achieve the downstream tasks. DefRec [37] introduced a new family of pretext tasks that support domain adaptations between different point-cloud datasets.

A key goal of these self-supervised models is to enable the network to learn valuable semantic information from large datasets during pretraining to assist downstream tasks. The purpose of this study is to pretrain using a task dataset to constrain the network so that it learns semantic and shape information, thereby improving performance.

### 2.3. Point-Cloud Reconstruction Models

In 2018, FoldingNet [13] was the first folding-based autoencoder. It is based on a graph neural network, and its decoder folds the grid twice to complete point-cloud reconstruction. This method effectively extracts point-cloud features and achieves unsupervised classification tasks. To better fit the surface of the reconstructed object, Groueix proposed multiple grids [22]. Deprelle used a combination of elementary structures to generate reconstructed point clouds [23] using point translation and patch deformation learning modules. PCN [20] uses a fold-based approach to achieve point-cloud completion tasks via the reconstruction of one sparse and one dense point cloud. In 2020, the shuffle attention network (SA-Net) [38] used a skip-attention to connect the encoder and decoder to allow the network to better utilize the symmetry and similarity of data. Additionally, the folding block proposed by this method can gradually generate complementary point clouds. Wang used a coarse-to-fine strategy to achieve point-cloud completion and upsampling tasks simultaneously [19].

In 2D deep learning, generative adversarial networks (GANs) can generate realistic images. For point clouds, GANs can generate new point clouds with improved quality. As the distribution of point-cloud data differs from that of 2D image data, a 3D GAN cannot be directly extended from 2D data. To overcome this, in 2018, the point-cloud GAN [39] first obtained the point distribution in the latent space using the autoencoder, and it used the generator and decoder parts to jointly realize its GAN function. The point-cloud upsampling GAN [40] generates dense and uniform point clouds, and the point fractal network [18] applies a GAN strategy to extract and mix the multiscale features of a point cloud using three-step downsampling followed by point-cloud reconstruction using three-step upsampling.

These reconstruction networks can all be combined with the point-cloud classification network after being properly tuned to enhance its performance. However, it requires careful design to properly couple them.

## 3. Our Method

In this section, we describe our self-supervised model, which improves the classification performance of the original network without introducing additional data. Then, we introduce the structure of the multigrid autoencoder, which is an important part of the self-supervised model. We also present the overall structure of the classification model, which slightly differs from PointNet++.

### 3.1. Self-Supervised Model

The combination of shared MLPs and max-pooling offers a simple solution to the disorderly nature of point clouds. However, building a classification network using only this method encounters a problem in that the extracted features are not rich enough to prevent information loss.

Our motivation for using the self-supervised method is to avoid the loss of shape information by pretraining the autoencoder, assuming that a well-designed autoencoder exists that can efficiently extract semantic information to perfectly reconstruct the point cloud. This autoencoder must be able to extract all point-cloud information, which enables the network to achieve its classification task. Notionally, pretraining the network with this autoencoder reduces its reliance on labeled data.

The new self-supervised framework is shown in Figure 1. The model consists of pretraining and classification parts. In the pretraining part, the task of the autoencoder is to make the output look the same as the input so that the encoder can convert the point-cloud information into high-dimensional features ingestible by deep learning, simplifying the classification task. Under ideal, well-designed conditions, the model extracts all point-cloud features if the input to the autoencoder is the same as the output. Thus, we only need to freeze the encoder and train the classifier alone to perfectly implement the classification task. However, in practical applications, the entire classification network must still be fine-tuned to avoid shape information loss.

Briefly, our self-supervised pipeline operates as follows. First, the autoencoder, consisting of an encoder and a multigrid decoder, is trained. Then, the classification network, which consists of the classifier and the frozen encoder, is trained. Finally, the entire classification network is fine-tuned.

### 3.2. Multigrid Autoencoder

On the one hand, our multigrid autoencoder can learn the shape information of point clouds; on the other, it might also learn meaningful semantic information.

**Figure 2:**
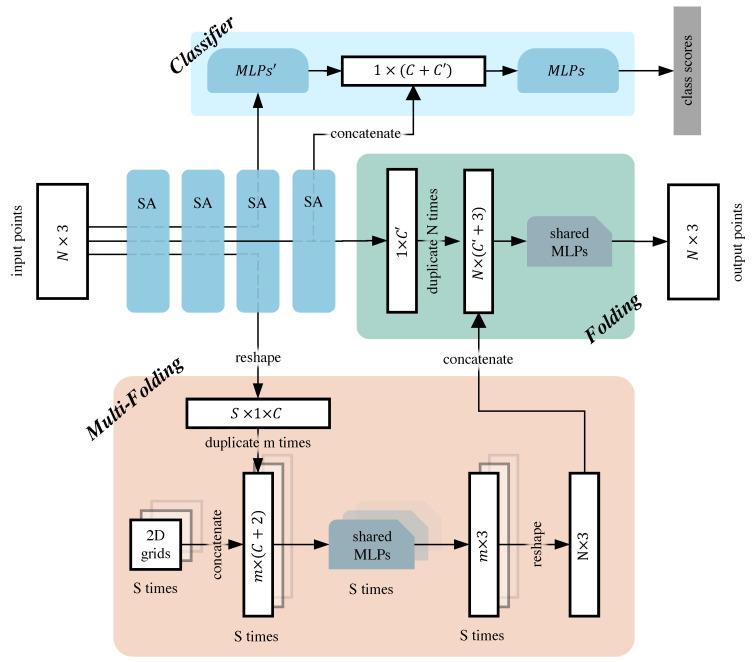
Overall framework of our model. Our proposed multigrid autoencoder consists of four self-abstraction (SA) modules with folding and multi-folding operations (see the second and third rows in the figure). Our proposed classification network consists of four SA modules and the classifier. For multi-folding, the shared multilayer perceptrons (MLPs) are three-layered, whose neurons are set to 514-256-64-3. For folding, the shared MLPs are also three-layered but set to 1027-256-64-3. The activation function is LeakyReLU, and batch normalization is added between each layer. Regarding the classifier, the MLPs’ consist of one MLP layer, and the variation in the number of neurons is S×C-512. There is also a three-layer MLP, whose variation in the number of neurons is 1536-512-128-k, where k represents the number of categories. Batch normalization and dropout are added between layers, and the activation function is LeakyReLU. Note that the solid line in the SA indicates that it is processed by the layer, and the dashed line in the SA indicates that the layer is skipped.

Our proposed autoencoder is shown in the second and third line of Figure 2. Its encoder consists of four PointNet++ set abstraction (SA) layers [8], and its decoder consists of multi-folding and folding operations. After a point cloud is an input to the encoder, it traverses multiple SA layers to extract features and then becomes input to the multi-folding and folding operation. The features of the input to the multi-folding operation are transformed by the point cloud through the first, the second, and the third layer SA. The features of the input to folding operation are transformed by the point cloud through the first, the second, and the fourth layer SA. The number of points in the encoder process changes as follows: *N*, 512, 128, S, 1, where *N* is the total number of points, and *S* is a variable.

The SA applies the farthest-point sampling algorithm so that the sampling points evenly cover the entire surface of the object. The ball query algorithm used by SA [8] effectively aggregates the information of neighboring points. Therefore, the *S* points output by the third layer of the encoder can cover the object evenly, and the features of each point are aggregated from those of the multilevel neighborhood points. The point output by the last layer of the encoder contains the global features of the point clouds. This means that the features fed into the multi-folding operation each contain the corresponding local features. The features that are fed into the folding operation are the global features of the point cloud.

Our proposed decoder, like FoldingNet, is divided into two folding operations. The multi-folding module is based on AtlasNet, which helps reconstruct the point cloud for its second folding operation. The structure of the folding operation is the same as that of the FoldingNet decoder’s folding operation.

The input to the multi-folding operation includes *S* 2D grids and the features of multiple point-cloud local regions extracted by the encoder which are the output of the third layer SA. Here, the 2D grid refers to a set of 2D points $P_{2Dgrid}\in R^{m\times 2}$ on the origin, consisting of the coordinates of a flat square grid with the number of points $m=n^{2}$, where *n* is the total number of edge points of the 2D grid, and N=S×m should also be satisfied. In addition, these 2D grids should be exactly the same. Feature $F_{in1}\in R^{S\times C}$ from the third layer SA is the input to the multi-folding operation, where *C* is the channel size. We duplicate $F_{in1}$*m* times to obtain the feature $F_{in1dup}\in R^{S\times m\times C}$. $F_{in1dup}$ is separated, then concatenated with *S*$P_{2Dgrid}$ to obtain *S* features $F_{fwg1}\in R^{m\times (C+2)}$. Next, *S*$F_{fwg1}$ will be processed by *S* shared-MLPs to obtain *S* output results $P_{out1^{\prime }}\in R^{m\times 3}$. Note that the parameters between *S* shared-MLPs are not shared. The settings for shared-MLPs can be found in the legend of Figure 2. Then, we concatenate *S*$P_{out1^{\prime }}$ together and reshape it to obtain a result $P_{out1}\in R^{N\times 3}$. In other words, the multi-folding operation performs *S* folding for the *S* local features of the point cloud and concatenates the results together as output.

**Definition** **1.** *We call the operation of folding multiple features from a point cloud multiple times and concatenating the results together a multi-folding operation.*

The global feature $F_{in2}\in R^{1\times C^{\prime }}$ from the fourth layer SA is the input of the folding operation, where $C^{\prime }$ represents the channel size. After $F_{in2}$ is duplicated *N* times, we obtain feature $F_{in2dup}\in R^{N\times C^{\prime }}$. Next, $F_{in2dup}$ is concatenated with $P_{out1}$ in the channel dimension, and we obtain $F_{fwg2}\in R^{N\times (C^{\prime }+3)}$. Finally, $F_{fwg2}$ is processed by shared-MLPs to obtain the reconstructed point cloud $P_{out2}\in R^{N\times 3}$.

Multigrid autoencoder uses the multi-folding module to perform the first folding operation of the local features output from the encoder, and then, it uses the folding module to perform the second folding operation of the global features output from the encoder. The two folding initialization point clouds are multiple 2D grids and the output from the multi-folding operations, respectively.

Due to the disorder of the point cloud, the loss function of the multigrid autoencoder needs to satisfy the permutation invariance. The commonly used loss functions for point-cloud reconstruction networks are Chamfer distance (CD) and earth mover’s distance, which both satisfy the permutation invariance. Compared with CD, the calculation of earth mover’s distance is more complicated, which results in a long calculation time. Therefore, like FoldingNet, we choose efficient CD as the loss function for the multigrid autoencoder.

(1) $$CD\left(X,Y\right)=\frac{1}{\left|X\right|}\sum_{x\in X}\min_{y\in Y}{∥x-y∥}_{2}^{2}+\frac{1}{\left|Y\right|}\sum_{y\in Y}\min_{x\in X}{∥x-y∥}_{2}^{2},$$

where *X* and *Y* are point clouds, and *x* and *y* are points.

### 3.3. Classification Model

Note that the pretrained and non-pretrained models exhibit different properties. For example, after pretraining, the output of the third SA module contains more valuable local features of the point cloud. Presumably, the classification network can utilize both local and global features.

To better couple the classifier and the multigrid autoencoder, we made some changes to the PointNet++’s classification network. The framework of our classification network is shown in Figure 2. The output of the third SA layer is *S* (i.e., extremely abstract local features containing almost all of the point-cloud information). We first use the MLPs to blend these features; then, we concatenate them with the global feature outputs from the fourth SA layer. Finally, we use MLPs to implement the classification task.

The original structure of the classification network in PointNet++ [8] was: three SA layers as an encoder and MLPs as a classifier. In contrast, our classification network uses four SA layers as encoders and two MLPs as classifiers. MLPs complete the fusion of local features and global features on point clouds.

## 4. Experiments

We first evaluated the self-supervised model using two datasets. Then, we evaluated the ability of multigrid autoencoders to extract semantic information from the point cloud. Thereafter, we demonstrated the effectiveness of the proposed classification network with ablation experiments. Then, we tested the pretraining method in a semi-supervised state. Finally, we assessed the relationship between the number of grids and model performance, and we visualized the point cloud to show how the grids comprised it.

### 4.1. Implementation Details

#### 4.1.1. Datasets

The ModelNet40 [41] dataset contains a total of 12,311 computer-assisted design models across 40 categories. They are split into 9843 models for training and 2468 for testing. As these data were generated from simulations, they have uniform densities with well-formed shapes. Achieving classification tasks on this dataset is relatively easy for networks.

PB_T50_RS in the ScanObjectNN [42] dataset contains a total of 14,298 objects across 15 categories. They are split into 11 416 objects for training and 2882 for testing. As these data were obtained by scanning real objects, and the authors made special perturbations to them, they are characterized by incompleteness and non-uniform densities. Achieving classification tasks on this dataset is challenging for most networks.

#### 4.1.2. Training Details

We constructed the networks using PyTorch on NVIDIA RTX3090 hardware. The number of points input to the network was 1024. Unless otherwise specified, we set the grid number to S=16. We used Adam as the optimizer and the initial learning rate to 0.01 with a decay factor of 0.8, occurring every five epochs until 0.00001. The batch size was set to 32. While training the multigrid autoencoder on the ModelNet40 dataset, we performed normalization, rotation, and jitter operations on the data. For the ScanObjectNN dataset, we performed only rotation and jitter operations.

During pretraining, we trained the autoencoder for 200 epochs. During the training phase of classification, we first froze the parameters of the encoder and fine-tuned the overall network after 10 epochs. To train the ModelNet40 dataset, we performed normalization and jitter operations. For the ScanObjectNN dataset, rotation and jitter were applied.

### 4.2. Classification Performance

The experiments mentioned in this subsection were carried out as follows. First, the network was pretrained with either ModelNet40 or ScanObjectNN, the classifier was trained, and overall fine-tuning was performed. Note that we did not introduce new data during pretraining.

The results of our proposed self-supervised method on ModelNet40 are presented in Table 1. The overall accuracy (OA) of the pretraining method on the ModelNet40 dataset was 92.7%, and the mean class accuracy (mAcc) was 89.9%. This result improves OA by 2% over PointNet++, demonstrating that our proposed pretraining method is significantly effective. Compared with PointWeb and SNR-PointNet++, which constrain PointNet++ to learn neighborhood information, our proposed model was 0.4% (OA) higher than PointWeb and 1.2% (OA) higher than SNR-PointNet++. Moreover, our method achieved better results than most previously proposed models.

ScanObjectNN is a recently built dataset. The results of its pretraining are presented in Table 2, where its OA is shown to be 82.6%, and the mAcc is 79.8%. Compared with extant networks, we outperformed most in OA and mAcc. This result improves OA and mAcc of PointNet++ by 4.7% and 4.4%, respectively.

There is still room for improvement in our method. Future improvement work should start from three aspects: enhancing the ability of the encoder to extract information, improving the reconstruction ability of the decoder, and coupling the encoder and decoder.

### 4.3. Classification Results on a Linear Support Vector Machine (SVM)

To determine whether feature vectors are meaningful, it is common to use them to train a linear SVM model under supervision and report its OA. To evaluate the multigrid autoencoder’s ability to extract semantic information from point clouds, we conducted the following experiments. First, we trained the multigrid autoencoder using the training set without supervision. Then, we trained three linear SVMs under supervision. Their inputs included the feature vector output by the third-layer SA, the output by the fourth-layer SA, and their concatenation. The test results are shown in Table 3. These experiments used the ModelNet40 or ScanObjectNN. All models listed in Table 3 used linear SVM classification after unsupervised training.

The ’third’, ’fourth’, ’third and fourth’ items in Table 3, respectively, represent the use of the feature vector output by the third layer SA, the output by the fourth layer SA, and their concatenation as the input to the SVM linear classifier.

As seen in Table 3, among the results of ModelNet40, that of ’third and fourth’ was the highest, indicating that it is reasonable to design a classifier using the outputs of the third- and fourth-layer SAs simultaneously. In this experiment with ModelNet40, our best result was 90.0%, which demonstrates that the network can extract valuable semantic information without supervision. This result exceeds those reported by FoldingNet, which originally justified the design of a multigrid autoencoder.

None of the results for ScanObjectNN in Table 3 exceeds 50%. This is because ScanObjectNN is a very challenging dataset for reconstruction tasks, and FoldingNet and multigrid autoencoder do not perform well on it. This shows that the classification network cannot directly use the features of the multigrid autoencoder output to complete the classification task; therefore, fine-tuning the overall network is necessary. It is worth noting that even in this case, the result of ’third and fourth’ is still the highest, which suggests that mixing the features from the third and fourth layers is reasonable after pretraining.

**Figure 3:**
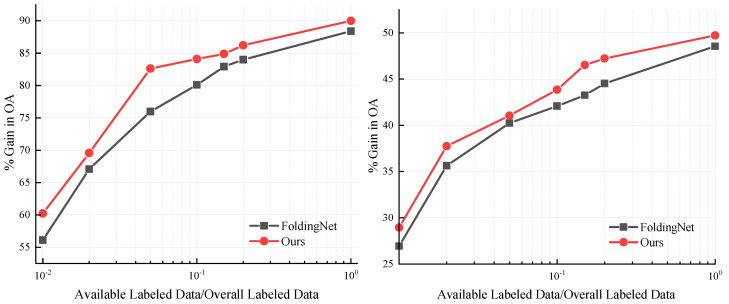
Test results of a linear support vector machine (SVM) classifier on ModelNet40 (**left**) and ScanObjectNN (**right**) under semi-supervised learning.

Figure 3 shows the test results of training a linear SVM classifier using only a portion of the supervised data after pretraining. In this experiment, the input to the SVM classifier was the concatenation of the feature vectors output by the third- and fourth-layer SAs. The red curve in the figure is the test result of the multigrid autoencoder, and the dark green curve is that of the FoldingNet. In the experimental results of ModelNet40 (Figure 3 left), the red curve is always above the dark green curve with a difference of 6.6% between their most distant points. In the experimental results of ScanObjectNN (Figure 3 right), the red curve is still always above the dark green curve. These results justify the design of the multigrid autoencoder.

### 4.4. Ablation Experiment

The results of the ablation experiments are presented in Table 4. ‘Pretrained’ in the table indicates that the classification network was pretrained with a multigrid autoencoder;.‘Third’ indicates that the output information of the third-layer SA of the classification network was used with the MLP. ‘Fourth’ indicates that the fourth-layer SA’s output was used. The experiments in this subsection used either ModelNet40 or ScanObjectNN for pretraining.

The structure of the baseline is as follows: the encoder is the 1st, 2nd, and 4th SA in Figure 2, the decoder is the FoldingNet’s decoder, and the classifier is the MLPs in Figure 2. That is, the baseline is the coupling of PointNet++ and FoldingNet using our proposed self-supervised framework. From Table 4, it can be observed that the OA of the baseline outperforms the OA of PointNet++ on both datasets. This proves that the self-supervised framework is helpful to improve the performance of the original network. It can also be observed that the OA of ‘pretrained+3rd+4th’ is outperforms than that of the baseline. This proves that multigrid autoencoder is more suitable for improving the performance of classification network than FoldingNet.

In the experimental results of ModelNet40, without pretraining, ‘3rd+4th’ is 1.6% and 0.7% higher than ‘3rd’ and ‘4th’, respectively. With pretraining, ‘3rd+4th’ is 2.7% and 1.8% higher than ‘3rd’ and ’4th’, respectively. With the help of multigrid autoencoder pretraining, the structure of mixed two feature vectors plays a more noticeable role. In the experimental results of ScanObjectNN, without pretraining, this structure does not seem to affect the results. With pretraining, ‘3rd+4th’ is 0.5% and 1.2% higher than ‘3rd’ and ‘4th’, respectively. This structure of two mixed feature vectors slightly improves the results.

In fact, the multigrid autoencoder does not perform as well on ScanObjectNN as it does on ModelNet40. Based on the above observations, we find that the effect of multi-scale fusion methods is easily affected by pretraining. When the classification network is not pretrained or the reconstruction effect of the pretrained network is not good, the effect of the multi-scale fusion method is not noticeable. Multi-scale fusion methods work better when the classification network is well pretrained.

### 4.5. Semi-Supervised Experiment

Our proposed autoencoder was unsupervised so we could enhance the encoder’s performance with unlabeled data during pretraining. In practical applications, obtaining data is not difficult, but labeling data is a tedious and lengthy task. Using unlabeled data to improve network performance is a very convenient task. We evaluated our pretraining method using a small amount of labeled data from ModelNet40 and ScanObjectNN datasets. The training process was as follows. First, we trained the autoencoder using the full training dataset; then, we used only a portion of the labeled data to train the classifier and fine-tune the overall network.

**Figure 4:**
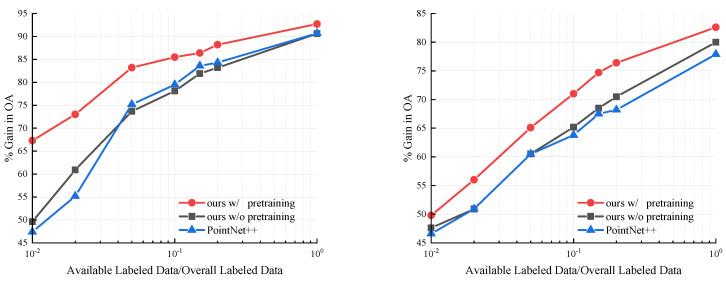
Evaluation results of training the network with a small amount of labeled data on ModelNet40 (**left**) and ScanObjectNN (**right**).

Figure 4 left and right show the evaluation results of ModelNet40 or ScanObjectNN. The red, dark green, and blue curves in the figure represent our proposed method with pretraining, ours without pretraining, and PointNet++, respectively. In multiple evaluations, we used labeled data comprising 1%, 2%, 5%, 10%, 15%, 20%, and 100% of the training dataset.

As illustrated in Figure 4 left, when the labeled data were reduced, our method with pretraining decreased more slowly than the other models. Additionally, the curve of the pretrained model remained significantly above that of the non-pretrained model, demonstrating that pretraining is effective. As illustrated in Figure 4 right the red curve is above the others. However, the gap between the red curve and the other two is not as significant as the gap in Figure 4 left, because the shape of the point cloud in ScanObjectNN was more complex, making multigrid autoencoder reconstruction very difficult. Nevertheless, Figure 4 right clearly shows the performance of our model on real-world point clouds. Pretrained networks perform better than non-pretrained networks with a small number of labeled samples because unsupervised autoencoders can learn more general features from the data. Non-pretrained networks are more prone to overfitting when trained only with a small number of labeled samples.

### 4.6. Effect of Grid on Classification Performance

**Figure 5:**
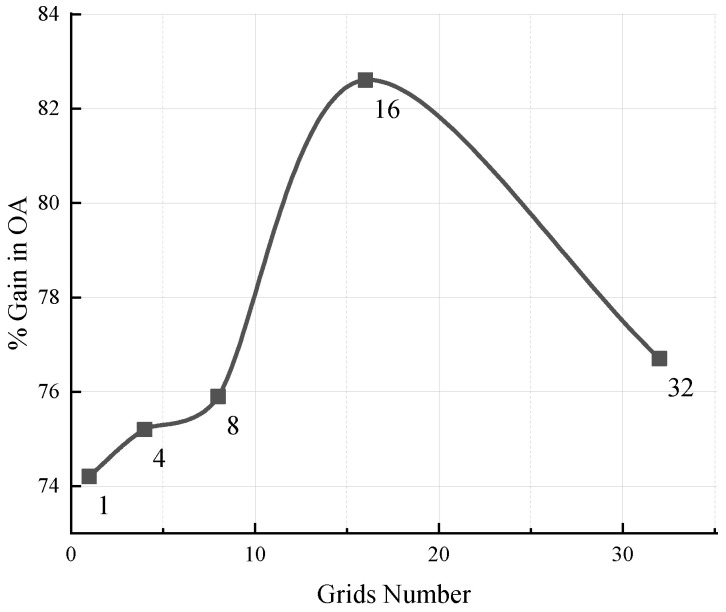
Evaluation results of the pretrained model using different numbers of grids (i.e., ScanObjectNN).

To better understand the model, we tested the self-supervised performance using different numbers of grids. Figure 5 shows the evaluation results of the model using ScanObjectNN with 1, 4, 8, 16, and 32 grids. The curve of Figure 5 rises throughout grids 1–16 and decreases from 16 to 32.

**Figure 6:**
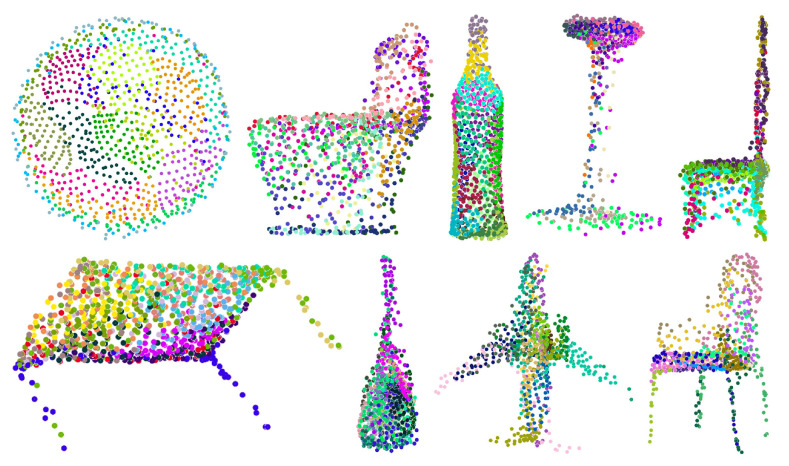
Visualization of point-cloud output by the multigrid autoencoder.

Assuming that the total number of points in the point cloud is *N*, and the set number of grids is *S*, each grid contains N/S points. After two folding operations of the multi-grid autoencoder, these grid points are mapped into the target point cloud. From the visualization results in Figure 6, it can be observed that the points within the same grid are still compact after mapping. That is, N/S points in a single grid are mapped to some area of the target point cloud. In the case of constant *N*, the larger the *S*, the smaller the area, and the smaller the *S*, the larger the area. We speculate that *S* affects the receptive field of the network. When *S* was small, the receptive field was larger, and when *S* was large, the receptive field was smaller. When the number of grids is moderate, that is, when the network has an appropriate receptive field, the performance of the network is optimal.

We conducted similar experiments on ModelNet40, where there were no big differences between experimental results corresponding to different grid numbers. Therefore, we only show the experimental results of ScanObjectNN.

### 4.7. Visualization

We visualized the point-cloud output from the multigrid autoencoder, as shown in Figure 6, using the ModelNet40 dataset. In the figure, each object consists of 16 different colored points, and those with the same color are from the same grid. From the objects in the first row and first column, the 16 grids were changed to form a bowl, and points from the same grid retained continuity. As seen with all objects, the 16 grids naturally divided the object into 16 parts.

## 5. Conclusions

In this work, we proposed the use of self-supervised methods to reduce information loss and the application of a multigrid autoencoder. The performance of the original network was effectively improved by using the multigrid autoencoder to pretrain the classification network. We conducted multiple experiments with PointNet++ as the test object, and the results demonstrate the effectiveness of our proposed self-supervised method. Notably, the multigrid autoencoder can extract meaningful shape information.

## Figures and Tables

**Table 1:** Classification accuracy on ModelNet40. ↑ represents the improvement compared to the baseline.

Methods	OA	mAcc
PointNet (2017) [7]	89.2	86.2
PointNet++ (2017) [8]	90.7	-
3DmFV (2018) [1]	91.4	-
Pointwise-CNN (2018) [43]	86.1	81.4
PointCNN (2018) [9]	92.2	88.1
PointGCN (2018) [44]	89.5	86.1
Moment (2019) [24]	92.4	90.3
MO-Net (2019) [24]	89.3	86.1
PointConv (2019) [27]	92.5	-
PointWeb (2019) [5]	92.3	89.4
SRN-PointNet++ (2019) [15]	91.5	-
DGCNN (2019) [45]	92.2	90.2
ConvPoint (2020) [10]	91.8	88.5
PointTransformer (2021) [14]	**93.7**	**90.6**
DFT-Net (2022) [46]	92.9	90.1
Our self-supervised method	92.7 (↑ 2.0)	89.9

**Table 2:** Classification accuracy on ScanObjectNN. ↑ represents the improvement compared to the baseline.

Methods	OA	mAcc
PointNet (2017) [7]	68.2	63.4
PointNet++ (2017) [8]	77.9	75.4
PointCNN (2018) [9]	78.5	75.1
SpiderCNN (2018) [47]	73.7	69.8
3DmFV (2018) [1]	63.0	58.1
DGCNN (2019) [45]	78.1	73.6
BGA-PN++ (2019) [42]	80.2	77.5
RPNet++ (2021) [48]	82.0	**79.9**
DRNet (2021) [49]	80.3	78.0
Hybrid-CNN (2021) [50]	80.7	-
PointNet++ w/ PatchAugment (2021) [51]	81.0	-
DGACN (2022) [52]	82.1	77.9
Our self-supervised method	**82.6** (↑ 4.7)	79.8 (↑ 4.4)

**Table 3:** Classification results on ModelNet40 and ScanObjectNN using a linear support vector machine (SVM).

Dataset	Methods	OA
ModelNet40	FoldingNet [13]	88.4
	Our third	87.7
	Our fourth	88.6
	Our third and fourth	**90.0**
ScanObjectNN	FoldingNet [13]	48.5
	Our third	42.8
	Our fourth	42.4
	Our third and fourth	**49.7**

**Table 4:** Ablation experiment results.

Dataset	Methods	Pretrained	Third	Fourth	OA
	Baseline	✓		✓	91.6
			✓		89.0
				✓	89.9
ModelNet40	Our		✓	✓	90.6
		✓	✓		90.0
		✓		✓	90.9
		✓	✓	✓	**92.7**
	Baseline	✓		✓	80.3
			✓		80.2
				✓	79.9
ScanObjectNN	Our		✓	✓	80.0
		✓	✓		82.1
		✓		✓	81.4
		✓	✓	✓	**82.6**

## Data Availability

Not applicable.
